# Analysis of two-decade meteorological and air quality trends in Rome (Italy)

**DOI:** 10.1007/s00704-022-04047-y

**Published:** 2022-04-14

**Authors:** Annalisa Di Bernardino, Anna Maria Iannarelli, Henri Diémoz, Stefano Casadio, Marco Cacciani, Anna Maria Siani

**Affiliations:** 1 Physics Department, Sapienza University of Rome, 00185 Rome, Italy; 2 SERCO SpA, 00044 Frascati, Rome, Italy; 3 ARPA Valle d’Aosta, Saint-Christophe, 11020 Aosta, Italy

## Abstract

This study presents a trend analysis of a two-decade (2000–2020) series of surface meteorological and air quality data measured in Rome (Italy). Data series are collected at three sites in Rome downtown and its coastal surroundings. This paper fills the gap due to missing or incomplete recent information about the meteorological and atmospheric composition tendencies in the Rome area. Datasets are subjected to in-depth quality control and to statistical analysis to verify the data homogeneity, whilst trend analysis is performed using the Seasonal Kendall test. The results show a statistically significant positive trend for average air temperature (0.07 °C year^−1^ in urban and coastal sites), whilst maximum and minimum temperatures increase more in urban (0.10 °C year^−1^) than in coastal (0.01 °C year^−1^) environment. The water vapour mixing ratio trend is higher in the city (0.10 g kg^−1^ year^−1^) than along the coast (0.03 g kg^−1^ year^−1^). The heat index tendency is more pronounced in the Rome centre (0.11 °C year^−1^) than in the coast (0.06 °C year^−1^). The monthly cumulative precipitations do not show statistically significant trends. On the other hand, air quality generally improved: surface pollutant concentrations show a significant decrease thanks to the reduction of local emissions (C_6_H_6_, − 0.12 µg m^−3^ year^−1^; SO_2_, − 0.09 µg m^−3^ year^−1^; CO, − 0.02 mg m^−3^ year^−1^; NO_x_, − 1.28 µg m^−3^ year^−1^; NO, − 0.38 µg m^−3^ year^−1^; NO_2_, − 0.60 µg m^−3^ year^−1^, PM_10_, − 0.35 µg m^−3^ year^−1^). Only O_3_ shows a statistically significant positive trend (0.15 µg m^−3^ year^−1^), in agreement with wider scale studies. The outcomes suggest that whilst local and national policies contribute to the improvement of air quality, the rising of temperature—as likely consequence of climate change—may be an emerging cause of concern for human thermal discomfort and potential effect on the conservation of tangible immovable heritage.

**Supplementary Information:**

The online version contains supplementary material available at 10.1007/s00704-022-04047-y.

## Introduction

Urban areas represent nowadays only 1.2% of land surfaces (Shepherd 2005) but they host about 55% of the global population (UN 2018) and most of the industrial sites. In the last decades, particular attention has been paid to two research topics concerning the atmosphere over urban areas: climate change and air pollution, together with their mutual interactions. The meteorological and chemical properties of the urban atmosphere are subject to the combined effects of local circulation and pollutant emission patterns, which are in turn affected by climate change and economic development. This urges for a thorough characterisation of local air quality that simultaneously accounts for the direct impact and the mutual interactions of all potential drivers, over a wide spectrum of temporal and spatial scales. To correctly evaluate the main characteristics of the urban atmosphere, several coexisting phenomena need to be analysed, including the Urban Heat Island (UHI) and the Urban Pollution Island (UPI), which are closely related (Ulpiani 2021) and result from the evolution of human settlement patterns induced by enhanced population growth and rapid urbanisation during the twentieth century (Li et al. 2020). As a matter of fact, urban sustainability concerns often resulted in limiting urban sprawl by resorting to urban densification and increased building heights (Borck 2016). The absorption of solar radiation from concrete surfaces is thus enhanced, and ventilation is potentially reduced due to the creation of artificial urban canyons (Kubilay et al. 2017; Di Sabatino et al. 2020). The intensive building is also often accompanied by the loss of temperature-mitigating green areas, yet with ambivalent effects on air pollution, depending on a variety of factors (Abhijith et al. 2017). Therefore, the response of any urban area to climate change needs to be assessed via the city- or region-specific characterisation of a variety of meteorological and chemical parameters. Filling this knowledge gap would effectively support decision-making in the contexts of industrial activity planning, urban design, environmental risk reduction and social welfare maximisation (Vogel and Henstra 2015). Another key aspect concerns the influence of climate and airborne pollutant stressors on cultural heritage, considering the impacts that may exist on various factors affecting the lifespan and durability of architectural heritage materials, elements and buildings (Dastgerdi et al. 2019). In addition, to evaluate climate-change effects and to plan the appropriate mitigation strategies, long-term, accurate and reliable data series are necessary (WMO 2017) but these datasets are not frequently available.

In Italy, the climatological features and the effects of local phenomena, such as the UHI, have been extensively examined in recent years (Mariani et al. 2016; Curci et al. 2021). Rome, the most populous and extended Italian city and one of the most densely populated in Europe (ISTAT 2021), has been studied since the 1980s, to evaluate the intensity and temporal variability of the UHI (Colacino and Lavagnini 1982; Bonacquisti et al. 2006). Nevertheless, a comprehensive analysis of the current trends of both meteorological and chemical characteristics of Rome’s atmosphere has not been carried out, yet. To fill this gap, this work analyses two decades of meteorological and in situ air quality data collected and managed by different national research institutes, such as (i) to provide a baseline to be used as a benchmark for alternative present climate observations and as a reference for future measurements; (ii) to evaluate the tendency of environmental conditions in the metropolitan area of Rome and the neighbouring coastal area and (iii) to support local policymakers in the optimal planning of climate-change mitigation strategies.

The paper is organised as follows: Sect. 2 describes the area of interest and introduces the in situ meteorological and air quality datasets, together with the data pre-processing. The results of the climatological investigations and the statistical analysis are presented and discussed in Sect. 3. Finally, Sect. 4 summarises the main outcomes of the study.

## Data and methods

### Site description

**Fig. 1:**
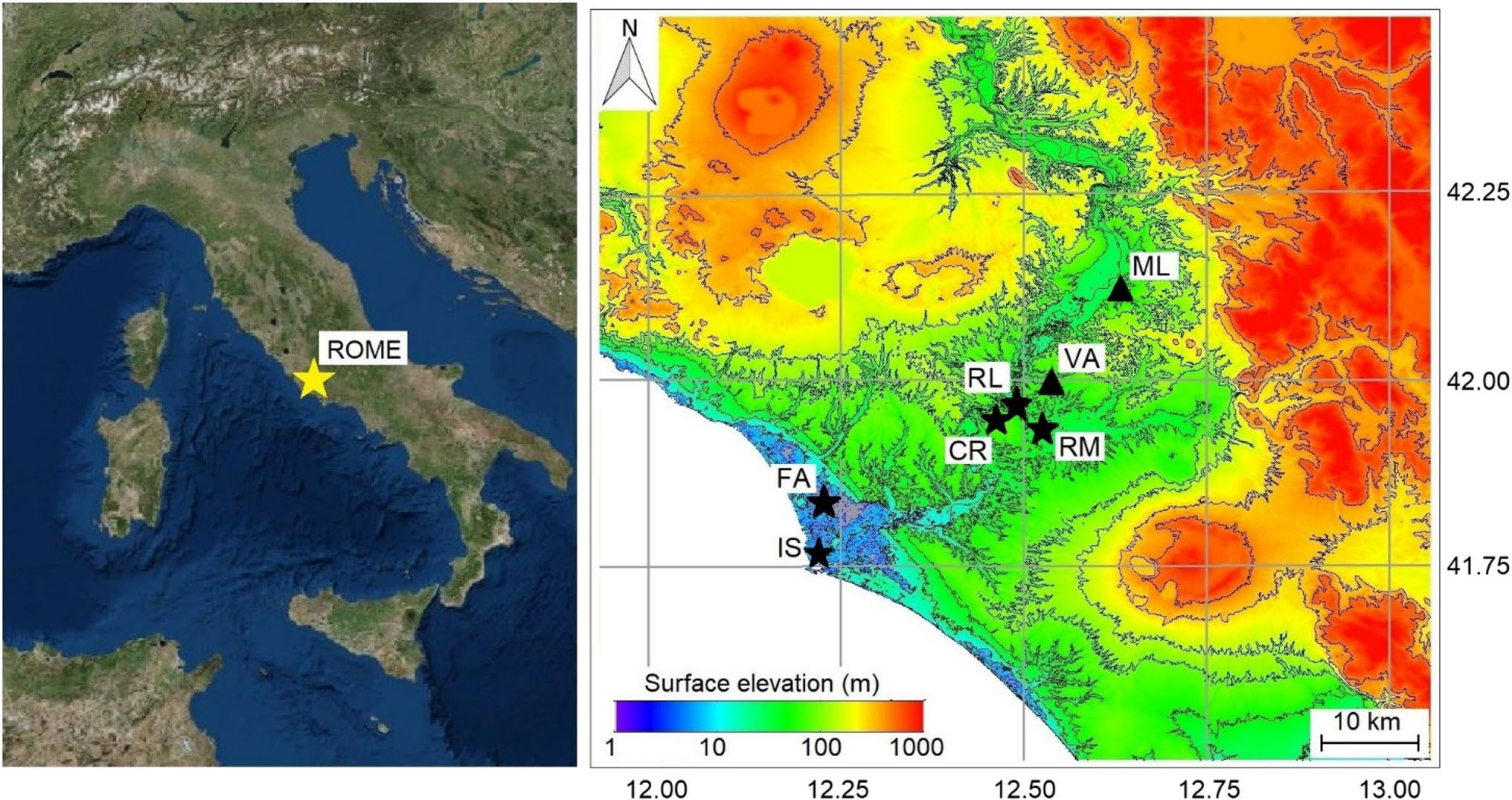
Geographical location (left, source: Google Earth) and Shuttle Radar Topography Mission (SRTM) map of the area investigated (right). The yellow star in the left panel identifies the location of the city of Rome; black stars and the black triangle in the right panel depict the location of the ground-based meteorological (Collegio Romano, CR; Roma Macao, RM; Roma Lanciani, RL; Fiumicino Airport, FA; Isola Sacra, IS) and air quality (Villa Ada, VA; Montelibretti, ML) stations, respectively

Rome is the capital of Italy, located in the central region of the Italian peninsula (Fig. 1, left panel). The metropolitan area covers about 1300 km^2^, where highly urbanised zones alternate with parks and green areas. The main activities are related to the services sector (ISTAT 2018); there are no relevant industrial plants and, therefore, the air quality is strongly influenced by local emissions (transport, domestic heating) and by advective phenomena (Gobbi et al. 2017).

As shown in the right panel of Fig. 1, the city extends along the Tiber valley (average altitude 21 m above sea level, m.a.s.l.) and is bounded by hills. Two prevailing wind patterns can be identified: the drainage flow through the Tiber valley (Petenko et al. 2011) and the sea/land breeze regime (Di Bernardino et al. 2021) that gives rise to Southwest winds during the day and Northeast winds at night. According to the Köppen-Geiger climate classification, Rome belongs to the Mediterranean climate class (Csa), i.e. temperate climate, with dry and hot summer (Beck et al. 2018). The local circulation is strictly connected to the synoptic high/low circulation systems over the Central-Eastern Mediterranean basin and Continental Europe.

### Datasets

In the following, a brief description of the stations and datasets used in the present study is provided. The right panel of Fig. 1 depicts the positions of the weather and air quality stations (black stars and triangle, respectively), whilst Table 1 summarises the locations of the stations, together with the variables provided and their availability time intervals.

**Table 1:** List of the stations considered in the present study and measured variables

Station	Coordinates	Altitude (m.a.s.l.)	Environment	Variable	Time interval
Collegio Romano (CR)	41.90° N, 12.48° E	57	Urban (rooftop)	T_ave_, T_min_, T_max_, RH, TP	2000–2020
Roma Macao (RM)	41.91° N, 12.51° E	84	Urban (rooftop)	T_ave_, T_min_, T_max_, TP	2000–2020
Roma Lanciani (RL)	41.92° N, 12.52° E	26	Urban (rooftop)	RH	2005–2020
Fiumicino Airport (FA)	41.80° N, 12.24° E	3	Coastal (ground)	T_ave_, T_min_, T_max_, RH	2000–2020
Isola Sacra (IS)	41.76° N, 12.23° E	4	Coastal (ground)	TP	2000–2020
Villa Ada (VA)	41.93° N, 12.51° E	50	Urban background (ground)	C_6_H_6_, SO_2_, CO, NO_x_, NO, NO_2_, O_3_, PM_10_	2000–2020
Montelibretti (ML)	42.06° N, 12.38° E	48	Rural background (ground)	O_3_	2000–2014

#### Meteorological datasets

To provide an in-depth picture of the climatological conditions and their temporal tendency together with their impact on thermal comfort and on cultural heritage, all the available long-time series of daily-averaged meteorological variables, collected both in urban and coastal environments, have been selected: air temperature (T_ave_, expressed in °C), relative humidity (RH, in %) and total precipitation (TP, in mm). In addition, minimum (T_min_, in °C) and maximum (T_max_, in °C) daily air temperatures are considered, in accordance with IPCC (2021) findings, which stressed the importance of extreme event analysis.

Based on the previous variables, it is possible to calculate composite indices, which turn out to be useful for specific purposes. For example, the water vapour mixing ratio (MR, in g kg^−1^), retrieved from RH and T_ave_, is a natural conservative gas tracer in atmospheric processes that do not involve condensation or evaporation (Wallace and Hobbs 2006). Here, MR is computed using a fixed reference value of 1013 hPa for the atmospheric pressure because of the lack of trustable time series of this variable (Camuffo 2010).

A further considered parameter is the heat index (HI, in °C), a bioclimatic parameter that combines air temperature and relative humidity to provide a human-perceived skin temperature (Steadman 1979). The National Oceanic and Atmospheric Administration (NOAA) classified the thermal discomfort level based on the value assumed by HI (Anderson et al. 2013), and suggested 32 °C as a reference value for “caution” level, with possible fatigue after prolonged exposure and/or physical activity.

HI is computed using Eq. 1 (Awasthi et al. 2021):

1 $$HI=-42.379+2.04901523\bullet T+10.14333127\bullet RH-0.22475541\bullet T\bullet RH-0.00683783\bullet {T}^{2}-0.00481717\bullet R{H}^{2}+0.00122874\bullet {T}^{2}\bullet RH+0.00085282\bullet T\bullet R{H}^{2}-0.00000199\bullet {T}^{2}\bullet R{H}^{2}+adjustment$$

where *T* is the air temperature in °F and *RH* is the relative humidity expressed as a percentage. The “adjustment” term (Lee and Brenner 2015) is defined as:

2 $$adjustment=\left\{\begin{array}{c}0 if T\le 80\wedge 0.13\le RH\le 0.85\\ \frac{13-RH}{4}\bullet \sqrt{\frac{17-\left|T-95\right|}{17}} if 80<T<112\wedge RH<0.13\\ \frac{RH-85}{10}\bullet \frac{87-T}{5} if 80<T<87\wedge RH>0.85\end{array}\right.$$

The weather stations considered in this study are listed and described below.

The Collegio Romano station (hereinafter, CR) is a historical meteorological observatory located in Rome downtown. The station, active since 1879, is located on the top of the tower of a historic building (Mangianti and Leone 2003) and is currently operated under the responsibility of CREA (Consiglio per la Ricerca in Agricoltura e l’Analisi dell’Economia Agraria, https://www.crea.gov.it/home). All the CR meteorological parameters used in the present paper (see Table 1) are acquired hourly, daily-averaged and refer to the period 2000–2020, i.e. since an automatic weather station (DA 9000, SIAP-MICROS, San Fior, Treviso, Italy) was installed at CR. The station provides the daily-averaged values of T_ave_, RH, the daily total amounts of precipitation and the daily values of T_min_ and T_max_.

The Roma Macao station (hereinafter, RM) is part of the Hydrographic and Sea Service network of the Lazio Region (Servizio Idrografico e Mareografico della Regione Lazio, https://www.idrografico.regione.lazio.it) and is located on a building rooftop in the urban centre of Rome. RM collects rainfall amount data and air temperatures using a 5-min interval. Here, the daily total amounts of precipitation, the daily-averaged mean temperature and the daily minimum and maximum air temperatures are considered for the period 2000–2020. The thermo-pluviometric data series are acquired following the WMO guidelines for meteorological instruments and methods of observation (WMO 1996).

The Roma Lanciani (hereinafter, RL) weather station belongs to the Regional Agency for the Development and Innovation of Agriculture of Lazio (ARSIAL, http://www.arsial.it/arsial/) and is located on the roof of a building in the Rome city centre, approximately 2 and 4 km Northeast of RM and CR, respectively. RL provided the daily-averaged measures of RH since 2005, used here as a reference in the comparison between meteorological variables in the urban environment. Thanks to the proximity to RM and to the similar environment in which the stations operate, datasets provided by RM and RL are merged to enlarge the urban dataset and to verify the mutual homogeneity of the datasets themselves (see the next section).

The Fiumicino airport weather station (World Meteorological Organization, WMO code: 16,242, International Civil Aviation Organization, ICAO code: LIRF, hereinafter, FA) is located Southwest of Rome, close to the Tyrrhenian coast and is managed by the National Agency for Flight Assistance (ENAV). Here, hourly-averaged data of meteorological quantities collected at ground level from 2000 to 2020 are considered. Measurements are collected following the WMO guidelines for meteorological instruments and methods of observation (WMO 1996).

The daily total precipitation series of the Fiumicino site is obtained from the Isola Sacra station (hereinafter, IS) of the Municipal Energy and Environment Company (ACEA), belonging to the Hydrographic and Sea Service network of the Lazio Region. IS station is installed on the ground, about 3 km South of FA, and provides the daily total amount of precipitation from 2000 to 2020. Thanks to the proximity between the FA and IS stations, their datasets are considered complementary for the definition of a single coastal site. Data series are acquired following the WMO guidelines for meteorological instruments and methods of observation (WMO 1996).

#### Air quality dataset

For what concerns the analysis of air quality, the standard threshold concentrations are established by the European Directive 1999/30/EC (EU 1999) and the European Air Quality Directive 2008/50/EC (EU 2008). To examine the air quality level, benzene (C_6_H_6_), sulphur dioxide (SO_2_), carbon monoxide (CO), nitrogen monoxide (NO), nitrogen dioxide (NO_2_), ozone (O_3_) and particulate matter (PM_10_) are considered. In addition, the potential ozone O_x_, defined as the sum of nitrogen dioxide and ozone atmospheric amounts, has been derived. This parameter is particularly useful as it has been demonstrated (Kley et al. 1994; Kalabokas et al. 2000) that, in urban environments, this quantity should be conservative over time scale of minutes, since the large part of the local NO_2_ present in the troposphere is produced at the expense of O_3_ (Kley et al. 1994).

The urban in situ air quality data refer to the Villa Ada station (hereinafter, VA), belonging to the air quality monitoring network managed by the Regional Agency for Environmental Protection (ARPA Lazio). The station, located at the edge of an urban park and classified as urban background, is equipped with sensors for the measurement of C_6_H_6_ (AirTOXIC, Chromatotec Europa, Val De Virvee, France), PM_10_ (SWAM 5a Dual Channel Monitor, FAI Instruments, Fonte Nuova, Rome, Italy), SO_2_, nitrogen oxides (NO_x_), CO and O_3_ (100E, 200E, 300E and 400E, respectively; Teledyne API, San Diego, CA, USA). The calibration of the sensors is checked quarterly, as required by the Legislative Decree 30 March 2017 GU n. 96 (https://www.gazzettaufficiale.it/eli/id/2017/04/26/17A02825/sg) and by the Italian institute for environmental protection and research guidelines (ISPRA 2014), whilst the calibration is annual. Data cover the period 2000–2020 and are provided in terms of daily averages.

The air quality station chosen for the downtown is the only one able to provide data covering the period 2000–2020. Unfortunately, no air quality data are available for the selected period in the coastal area under investigation.

In addition, the Montelibretti station (hereinafter, ML) was chosen as rural reference for the evaluation of the tropospheric contribution of O_3_ level. The station, located about 30 km northeast of Rome, is not influenced by local pollution sources and is managed by the Institute of Atmospheric Pollution of the National Research Council (CNR-IIA). ML was part of the European Monitoring and Evaluation Programme (EMEP) network until 2014. The average daily concentrations of ozone, monitored with a Dasibi UV photometric analyser mod. 1108 (Glendale, CA, USA), were extracted from the EMEP database, accessible online (Schultz et al. 2017), for the period 2000–2014.

### Data pre-processing and quality check

All the data used in this study have been previously validated by the institutional entities responsible for the data production and distribution, following their specific procedures (see details in Sect. 2.2). Nonetheless, in this work, further pre-processing and quality control are performed. The procedure can be divided into three steps.

Firstly, datasets underpass a preliminary screening through visual inspection to remove gross errors (such as negative RH and TP values, T_min_ values greater than T_max_ for the same day, air pollutant concentrations less than zero).

Secondly, for the correct evaluation of the monthly average mass and volume concentrations and, therefore, of robust long-term analysis, the months during which the amount of daily data is less than 80% are discarded. This availability threshold was chosen following the WMO guidelines (2017), considering the period of activity of each station. The station with the highest percentage of missing data is IS, where TP time series does not exceed the threshold in 26.7% of cases. Excluding IS, the percentage of months available is always greater than 96.8%. Specifically, ML has 96.8% of acceptable ozone values, CR has 98.75% of available monthly averages (rejected data equally distributed on T_ave_, RH and, consequently, HI) and at the RL station, 99.7% of data are accepted, whilst at RL and VA, 99.8% of the monthly averages are usable. Lastly, at FA, 100% of the months analysed exceed the abovementioned threshold. In all the examined stations, the rejected values are randomly distributed throughout the year, with negligible effects on both the statistics and the temporal trends. After verifying the temporal coverage of the datasets, the monthly statistics are computed from the daily or sub-daily data: for the meteorological quantities, the arithmetic means of T_ave_, T_min_ and T_max_ and RH and the cumulative values of TP have been calculated for each month, whilst for the atmospheric pollutants, the monthly-averaged concentrations are computed.

Lastly, all meteorological monthly series are subjected to a homogenisation procedure to identify and remove non-climatological signals, e.g. instrumental artefacts (Manara et al. 2018; Cerlini et al. 2020). This procedure is particularly relevant for the correct evaluation of temporal trends and both direct and indirect methods are usually employed. The first group of methods is based on the use of metadata, which provides information on the history of the station (e.g. sensor calibration, prolonged malfunctions, refurbishment and displacement of the station) but is often insufficient to evaluate the actual homogeneity of the data. The second kind of method, employed in this study, is based on statistical techniques and uses available metadata only as support and confirmation of the results.

In the present work, the Craddock test (Craddock 1979), most suitable for analysing series belonging to different networks, is adopted. The use of such time series permits the identification of temporal inhomogeneities due to simultaneous changes on the entire network, otherwise not recognisable. The Craddock test allows for the comparison of each test series with a “reference” series, considered more reliable. In the absence of an a priori reference, in this work, any series is alternately considered as “reference”, i.e. the Craddock test was applied in a pairwise comparison of stations (Sanchez-Lorenzo et al. 2015). For each combination, the cumulative normalised differences between the test and “reference” series are computed. The breakpoints in the test series are identified by changes in the slope of the cumulative normalised difference series.

During the period 2000–2020, marked slope changes are identified only in the time series of RH measured at CR (in pairwise comparison with both FA/IS and RM/RL). Specifically, two abrupt changes can be identified in 2008 and 2010. Unfortunately, the metadata does not provide enough information to interpret the test results. Consequently, the RH and HI (dependent on RH) time series collected at CR will not be used for the atmospheric characterisation and the evaluation of the temporal trends. It is further emphasised that thanks to the proximity of the meteorological stations of RM and RL and of FA and IS, hereinafter, these pairs of stations are considered as single sites (namely RM/RL and FA/IS, respectively), thus providing a more complete picture of both the urban and the coastal environments.

### Trend analysis of atmospheric variables

To provide a quantitative analysis of inter-annual trends, the Seasonal Kendall test (SK test), proposed by Hirsch and Slack (1984), has been applied. It is a non-parametric test, which analyses data for monotonous trends with seasonal variability, unlike linear tests, de-seasonalising the datasets, and considering the annual variability of the variables. In recent years, this test has been used for environmental and climatological studies (e.g. Viola et al. 2014; Ahmad et al. 2014) and to evaluate the improvement/deterioration of air quality (Cattani et al. 2010; Yolsal 2016; Ravindra et al. 2020).

Assuming a significance level α = 5% (i.e. the null hypothesis of no-trend is rejected), the outputs provided by the test are Mann-Kendal’s score (S) and its variance (${\sigma }_{s}$), Kendall Tau (τ), normalised test statistics (Z), SK slope and intercept of Kendall-Theil Robust Line. The last four parameters allow for the evaluation of temporal trends.

Positive (negative) S values indicate a positive (negative) trend, whilst absolute values of S close to zero detect no trend. The value of $\tau$ points out the strength of the monotonic association between the variable under investigation and time. It ranges between − 1 and 1 and it is analogous to the correlation coefficient in regression analysis. Z is obtained from the normal distribution standard quantiles and permits to reject the null hypothesis of no trend if $Z\ge {Z}_{1-\alpha }$, where ${Z}_{1-\alpha }$ is the ${100\left(1-\alpha \right)}^{th}$ percentile of the standard normal distribution. Moreover, to estimate the significance of the trend, Hirsch and Slack (1984) proposed the SK slope estimator, which depicts the annual magnitude of the increasing/decreasing tendency, and the intercept of the Kendall-Theil Robust Line, calculated from the line running through the monthly median of the input data.

## Results and discussion

### Climatological characterisation of Rome

In this section, the main climatological features of the region of interest are discussed.

**Fig. 2:**
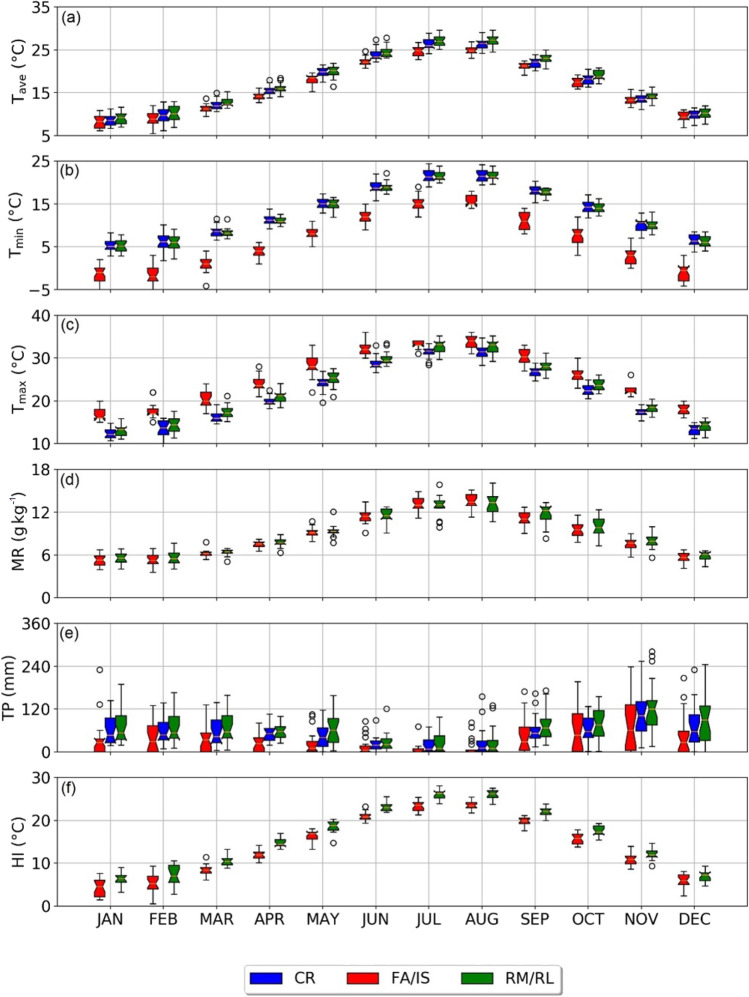
Monthly boxplot of multi-year **a** average air temperature, **b** minimum air temperature, **c** maximum air temperature, **d** mixing ratio, **e** total precipitations and **f** heat index collected at CR (blue), FA/IS (red) and RM/RL (green). Note the different ranges of the ordinate axis in panels **a**, **b** and **c**. Lower and upper whiskers depict the 25th and 75th percentiles, respectively; the notch shows the 95% confidence interval around the median and the open circles are the outliers

Figure 2 presents the multi-year box plot of meteorological parameters collected in the urban stations of CR, RM/RL and the coastal site of FA/IS. The average monthly values of meteorological variables, together with the annual mean, summarised in Table A1, are referred to as the average year of the period investigated.

The average temperatures (Fig. 2a) in the urban sites show the lowest values in January (8.6 °C and 9.1 °C at CR and RM/RL, respectively), whilst maxima are found during August (26.3 °C and 27.1 °C at CR and RM/RL, respectively). In the coastal area, the monthly T_ave_ values are always lower than in the city, ranging between 8.2 °C (January) and 24.8 °C (August). On average, CR and RM/RL differ by about 1.0 °C, with peaks during summer (2.3 °C in August) and minima differences during winter (0.2 °C in November and December). This dissimilarity may be related both to technical and physical reasons: in fact, the two stations are installed at different heights above the ground (CR and RM/RL are placed on buildings’ roofs, FA/IS on the ground), overlapping the combined effect of coastal cooling due to the sea breeze regime, more intense near the coast than in the city centre, and, above all, the UHI effect that affects Rome (Fabrizi et al. 2010).

The monthly behaviour of T_min_ and T_max_, depicted in Fig. 2b and c, respectively, shows that the minimum monthly temperatures are always higher in the city. The differences are attributable to the dissimilar altitude of the stations, to the UHI effect, contributing to increasing the minimum temperatures (Arnfield 2003), and to the canyon effect, responsible for limited ventilation close to the ground. The largest difference with respect to the coastal site is recorded in February (difference of about 8.0 °C on the medians), whilst the T_min_ differs less during summer (in August, difference between the medians of about 6.0 °C). On the contrary, the coastal site of FA/IS records the highest T_max_ throughout the year: it reaches a median value of 34 °C in August (in the same month, the median value is 31.5 °C at CR and 31.1 °C at RM/RL). Both in urban and coastal sites, the lowest T_max_ is observed during January (median T_max_ of 12.2 °C, 13.1 °C and 16.7 °C at CR, RM/RL and FA/IS, respectively). The difference between CR, RM/RL and FA/IS decreases in summer (in July, difference of approximatively 2 °C) and increases in autumn and winter (in December, 4.4 °C). The milder coastal climate, with lower annual temperature excursion and higher T_min_, is the result of the thermoregulatory action of the sea, which, in winter, gradually releases the heat absorbed in the warmer months by solar heating. In all the stations, the outliers are recorded mainly in spring and winter, although, especially in the latter case, they differ little from the percentile values (25th and 75th) shown in the graph.

The values of MR (Fig. 2d) are only available for RM/RL and FA/IS since the time series of RH measured in CR was excluded. Values are quite similar in the two sites, with a greater spread in the city. During winter, MR is approximately 5.5 g kg^−1^ for both sites, whilst during summer it reaches 16.1 g kg^−1^ and 15.1 g kg^−1^ at RM/RL and FA/IS, respectively, since MR is calculated starting from the data of T and RH, also MR assumes higher values during summer, when the amount of water vapour increases because of the higher evapotranspiration from wet surfaces and the higher frequency of convective events. The outliers are recorded mainly during spring and summer, although their values are very close to the percentiles, as shown in the figure.

The average annual cumulative rainfall, calculated over the 20 years, is 638.9 mm at CR, 780.3 mm at RM/RL and 440.9 mm at FA/IS. The monthly distribution of TP (Fig. 2e) shows that in both urban and coastal environments, the wettest month is November (103.2 mm, 126.7 mm and 78.8 mm at CR, RM/RL and FA/IS, respectively), whilst the month with the least rainfall is July (19.5 mm, 25.4 mm and 7.2 mm at CR, RM/RL and FA/IS, respectively). Rainfalls are concentrated in autumn and winter, whilst TP decreases during spring and reaches its minimum in summer. The presence of the outliers underlines that the area is subject to intense precipitations during both summer (when rainfall is often in the form of severe thunderstorms) and winter.

The HI behaviour (Fig. 2f) is, by definition, dependent on T_ave_ and RH (see Eq. 1). The minimum values are recorded in January (6.1 °C and 4.4 °C at RM/RL and FA/IS, respectively) and the maxima in August (26.1 °C and 23.5 °C at RM/RL and FA/IS, respectively). Also during the warm season both in Rome downtown and in its coastal surroundings, HI is always below the “caution” level of 32 °C (Anderson et al. 2013). It should be noted that the values are always higher in the urban area and that the physiological discomfort caused by the high temperatures and high humidity levels tends to increase during spring and summer. In addition, few outliers are identifiable in all seasons, with values very close to the percentiles.

### Temporal trends of meteorological data

**Fig. 3:**
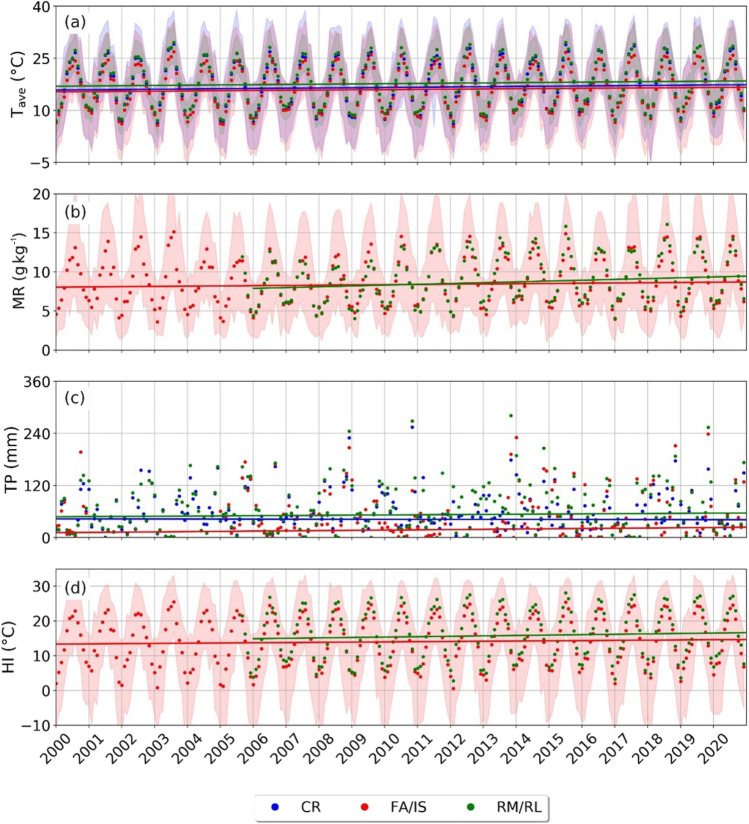
Monthly averages of **a** average air temperature at CR, FA/IS and RM/RL; **b** mixing ratio at RM/RL and FA/IS; **c** total precipitations at CR, FA/IS and RM/RL and **d** heat index at RM/RL and FA/IS. Where applicable, the shaded regions represent minima and maxima values. The solid lines depict the Kendall-Theil Robust Line, computed considering the slope and the intercept of the SK test for each parameter

Figure 3 shows the time series of the ground-based meteorological variables estimated at the urban and coastal sites. To provide a quantitative analysis of the climatic peculiarities of the sites and to minimise the effect of inter-annual fluctuations, the Seasonal Kendall test has been applied, allowing for a systematic statistical analysis with a seasonal adjustment method. Table 2 reports the test results for the CR, RM/RL and FA/IS sites. In addition, the SK test was also applied by splitting the time series according to the meteorological seasons (spring: March, April and May; summer: June, July and August; autumn: September, October and November; winter: December, January and February). The results are summarised in Table A2.

**Table 2:** Results of Seasonal Kendall test for the meteorological data collected both at the urban (CR, RM/RL) and coastal (FA/IS) sites. The values of Kendall correlation coefficient (τ), normalised test statistics (Z) and SK slope and intercept of Kendall-Theil Robust Line are presented. Slope and intercept are expressed following the original units of measurement of the parameters. In the trend column, ↑ (↓) indicates a positive (negative) statistically significant trend, whilst ↔ depicts no statistically significant trend. The variables with statistically significant trends are in bold

Variable	Site	τ	Z	Slope	Intercept	Trend
**T**_**ave**_	**CR**	**0.22**	**4.74**	**0.07 °C year**^**−1**^	**15.8 °C**	**↑**
	**FA/IS**	**0.20**	**4.47**	**0.06 °C year**^**−1**^	**15.3 °C**	**↑**
	**RM/RL**	**0.20**	**4.40**	**0.07 °C year**^**−1**^	**16.9 °C**	**↑**
**T**_**max**_	**CR**	**0.12**	**2.56**	**0.04 °C year**^**−1**^	**20.4 °C**	**↑**
	**FA/IS**	**0.13**	**2.98**	**0.01 °C year**^**−1**^	**25.0 °C**	**↑**
	**RM/RL**	**0.28**	**6.12**	**0.10 °C year**^**−1**^	**21.2 °C**	**↑**
**T**_**min**_	**CR**	**0.31**	**6.72**	**0.12 °C year**^**−1**^	**11.6 °C**	**↑**
	**FA/IS**	**0.11**	**2.42**	**0.01 °C year**^**−1**^	**5.5 °C**	**↑**
	RM/RL	0.07	1.54	0.02 °C year^**−**1^	12.4 °C	↔
**MR**	**FA/IS**	**0.15**	**3.18**	**0.03 g kg**^**−1**^** year**^**−1**^	**8.0 g kg**^**−1**^	**↑**
	**RM/RL**	**0.36**	**6.54**	**0.10 g kg**^**−1**^** year**^**−1**^	**7.8 g kg**^**−1**^	**↑**
**TP**	CR	− 0.02	− 0.47	− 0.08 mm year^**−**1^	43.1 mm	↔
	**FA/IS**	**0.23**	**5.02**	**0.57 mm year**^**−1**^	**10.8 mm**	**↑**
	RM/RL	0.05	1.16	0.40 mm year^**−**1^	47.6 mm	↔
**HI**	**FA/IS**	**0.21**	**4.51**	**0.06 °C year**^**−1**^	**13.3 °C**	**↑**
	**RM/RL**	**0.25**	**4.54**	**0.11 °C year**^**−1**^	**14.8 °C**	**↑**

The average air temperature (Fig. 3a) measured at CR ranges between 6.1 °C (February 2012) and 29.1 °C (August 2003) with a slightly positive trend, detectable also for T_min_ and T_max_. At RM/RL, the average air temperature in the last 20 years is between 6.9 °C (February 2012) and 29.6 °C (August 2003). In the FA coastal site, the temperature trends are comparable with those of the urban sites even if higher (lower) maximum (minimum) temperatures are evident. The tendencies are confirmed by the SK test: at CR, T_ave_, T_max_ and T_min_ are slightly increasing over the years with slopes equal to 0.07 °C year^−1^, 0.04 °C year^−1^ and 0.12 °C year^−1^, respectively. The largest increase is found for T_min_ (τ = 0.31 and Z = 6.72), followed by T_ave_ (τ = 0.22 and Z = 4.74) and T_max_ (τ = 0.12 and Z = 2.56). Otherwise, at RM/RL, only T_ave_ and T_max_ show a statistically significant increasing trend (τ = 0.20, Z = 4.40 and slope 0.07 °C year^−1^ for T_ave_; τ = 0.28, Z = 6.12 and slope 0.10 °C year^−1^ for T_max_), whilst T_min_ assumes almost constant values over the years (τ = 0.07, Z = 1.54 and slope 0.02 °C year^−1^). In the coastal site, all the thermal variables show a statistically significant increasing trend, more intense for T_ave_ (τ = 0.20, Z = 4.47 and slope 0.06 °C year^−1^) with respect to T_max_ and T_min_, whose trends are comparable (τ = 0.13, Z = 2.98 and slope 0.01 °C year^−1^ for T_max_; τ = 0.11, Z = 2.42 and slope 0.01 °C year^−1^ for T_min_). Seasonal analysis (Table A2) shows that for T_ave_, statistically significant upward trends are detectable during spring and summer (CR) and in summer and autumn (RM/RL and FA/IS) possibly linkable to a different thermoregulation effect of the sea in the various seasons. In the other seasons, although T_ave_ is increasing, no statistically representative trends are present. Otherwise, T_max_ exhibits increasing trends only at RM/RL (summer, autumn and winter) and T_min_ increases at CR (all seasons) and at FA/IS (summer). T_min_ is mainly affected by the radiative exchange from the ground, and it is considered a good fingerprint of the greenhouse effect due to the water vapour and other atmospheric gases and urbanisation (Langeron et al. 2020). Also in these cases, in the other seasons, the temperatures measured in the last 20 years are increasing without statistical significance.

The MR (Fig. 3b) average values are approximately 9.0 g kg^−1^ at RM/RL and 8.9 g kg^−1^ at FA (see Table A1). Considering the behaviour over the period under investigation, as already noted for the average monthly trend (Fig. 2), the presence of a seasonal cycle is evident: in summer, the values are higher, whilst minima are recorded in autumn and winter. Based on the SK test (τ = 0.36 and Z = 6.54 at RM/RL, τ = 0.15 and Z = 3.18 at FA/IS), a statistically significant increasing trend can be identified both in the urban and coastal sites, but the sloping trend is higher in Rome downtown (0.10 g kg^−1^ year^−1^) than along the coastline (0.03 g kg^−1^ year^−1^).

Total monthly rainfalls, TP, (Fig. 3c) also show a clear seasonal cycle: as expected in the case of the Mediterranean climate, rainfalls are more frequent in autumn, with a minimum in summer (Kelley et al. 2012). The dataset reveals that the record of total monthly rainfall at CR was measured during November 2010 with a maximum of 254.8 mm; at RM/RL, the maximum TP was 281.0 mm during November 2013 whilst at the FA/IS site, TP of 238.8 mm was collected during November 2019. The SK test shows a significant increasing trend over time only for the coastal site FA/IS (τ = 0.23, Z = 5.02 and slope 0.57 mm year^−1^) during spring, summer and autumn (see Table A2), whilst no annual trends are identifiable at CR (τ =  − 0.02, Z =  − 0.47 and slope − 0.08 mm year^−1^) and RM/RL (τ = 0.05, Z = 1.16 and slope 0.40 mm year^−1^).

As shown in Fig. 3d, the monthly average values of HI, computed at RM/RL and FA stations using Eq. 1, are always below 32 °C (i.e. it is always below the “caution” level established by the NOAA) and increase over years, as confirmed by the SK test (τ = 0.25, Z = 4.54 and slope 0.11 °C year^−1^ at RM/RL; τ = 0.21, Z = 4.51 and slope 0.06 °C year^−1^ at FA/IS). In both sites analysed, the growing trends are statistically significant in summer and autumn. Results suggest that, in the period 2000–2020, the physiological discomfort caused by high temperatures significantly increased.

Most of the meteorological variables show an appreciable positive trend over the years, and only some of them assume constant values in the investigated period. In detail, the parameters closely regulating the thermal comfort, i.e. T_ave_, T_max_ and T_min_, as well as HI, have a statistically significant positive trend both in urban and coastal environments (except for T_min_ at RM/RL). Moreover, rainfalls are constant in the urban sites and are slightly increasing only along the coastline, and T_max_ and HI are increasing, worsening the perception of summer temperatures, whilst the upward trend of T_min_ and T_max_, linkable to the high frequency of extreme weather events, suggests an increasing diurnal and nocturnal thermal discomfort. The results obtained in this study are in agreement with the recent report published by ISPRA (2021a, 2021b), in which the national trends of mean, minimum and maximum temperatures over the period 1981–2020 are calculated. Through the application of a linear regression model, statistically significant increasing trends for T_ave_ (about 0.04 °C year^−1^), T_min_ (about 0.03 °C year^−1^) and T_max_ (about 0.04 °C year^−1^) were identified over the Italian territory. The growth rates obtained in the present study for the Rome area show a slightly larger increase than the national average for temperatures (average value of the three sites of T_ave_ = 0.07 °C year^−1^, T_max_ = 0.05 °C year^−1^ and T_min_ = 0.06 °C year^−1^). Similarly, ISPRA (2021a, 2021b) states that the national and regional cumulative annual precipitation does not show statistically significant trends, in accordance with the findings of this study. In fact, only the FA/IS site during spring, summer and autumn shows a positive tendency, whilst in the two urban sites, there are no statistically significant trends. These outcomes can be both interpreted as indicators of the UHI effect and of climate change, which is affecting, albeit limitedly, also the urban area of Rome and its surroundings. The detected trends are only partially attributable to the transformations of the city, being Rome a metropolis with a well-developed building texture in the historical centre and with a limited recent architectural change and a dispersed and fragmented urban expansion (Bianchini et al. 2021).

### Temporal trends of air quality data

**Fig. 4:**
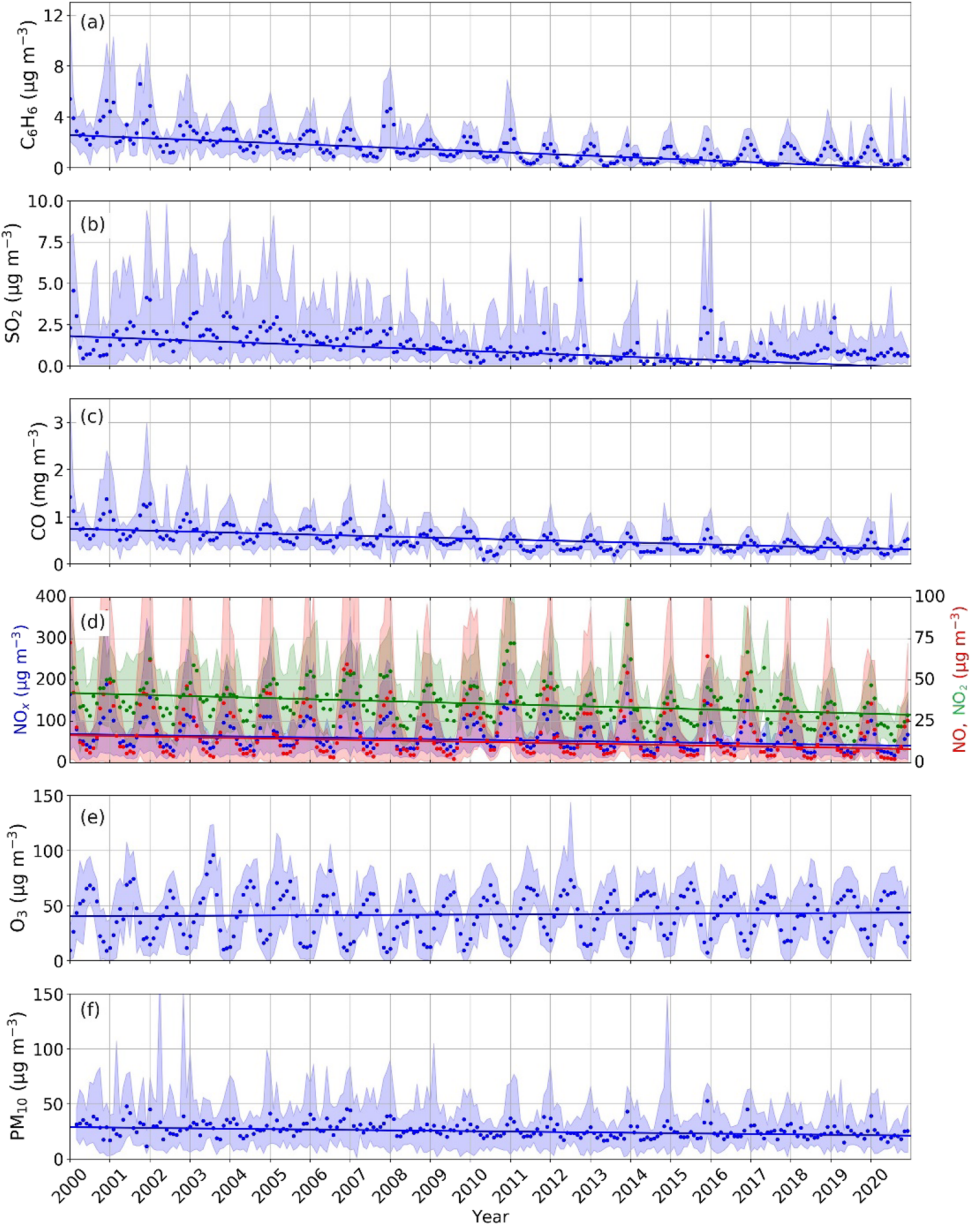
Trend of monthly average in situ concentrations of **a** benzene; **b** sulphur dioxide; **c** carbon monoxide; **d** nitrogen oxides, nitrogen monoxide and nitrogen dioxide; **e** ozone and **f** PM_10_ collected in VA station. The shaded regions represent minima and maxima values. The solid lines depict the Kendall-Theil Robust Line, computed considering the slope and the intercept of the SK test for each parameter

**Fig. 5:**
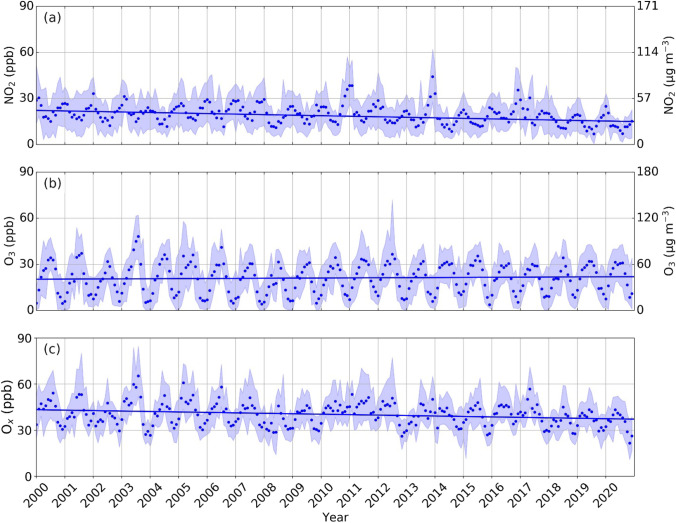
Trend of monthly average in situ concentrations of **a** nitrogen dioxide, **b** ozone and **c** potential ozone measured at VA. The shaded regions represent minima and maxima values. The solid lines depict the Kendall-Theil Robust Line, computed considering the slope and the intercept of the SK test for each parameter

Figures 4 and 5 show the time series of the monthly averaged concentrations retrieved from data provided by the ARPA Lazio air quality monitoring network over the period 2000–2020. Table 3 depicts the results of the SK test, whilst in Table A3, the seasonal results of the SK test are summarised.

**Table 3:** Results of Seasonal Kendall test for in situ air pollutants. Kendall correlation coefficient and slope are expressed as yearly rates of variation. Note that ΔO_3_, defined as the difference between urban (VA) and rural (ML) concentrations, and O_3_ at ML refer to the period 2000–2014. In the trend column, ↑ (↓) indicates a positive (negative) statistically significant trend

Variable	Site	τ	Z	Slope	Intercept	Trend
C_6_H_6_	**VA**	** − 0.73**	** − 16.46**	** − 0.12 µg m**^**−3**^** year**^**−1**^	**2.7 µg m**^**−3**^	**↓**
SO_2_	**VA**	** − 0.46**	** − 10.39**	** − 0.09 µg m**^**−3**^** year**^**−1**^	**1.9 µg m**^**−3**^	**↓**
CO	**VA**	** − 0.75**	** − 17.05**	** − 0.02 mg m**^**−3**^** year**^**−1**^	**0.8 mg m**^**−3**^	**↓**
NO_x_	**VA**	** − 0.45**	** − 10.30**	** − 1.28 µg m**^**−3**^** year**^**−1**^	**70.3 µg m**^**−3**^	**↓**
NO	**VA**	** − 0.45**	** − 10.17**	** − 0.38 µg m**^**−3**^** year**^**−1**^	**17.0 µg m**^**−3**^	**↓**
NO_2_	**VA**	** − 0.36**	** − 8.14**	** − 0.60 µg m**^**−3**^** year**^**−1**^		
				**(− 0.33 ppb year**^**−1**^**)**	**42.8 µg m**^**−3**^	
					**(22.6 ppb)**	**↓**
O_3_	**VA**	**0.11**	**2.46**	**0.15 µg m**^**−3**^** year**^**−1**^** (0.07 ppb year**^**−1**^**)**	**40.3 µg m**^**−3**^** (20.2 ppb)**	**↑**
O_x_	**VA**	** − 0.25**	** − 5.51**	** − 0.28 ppb year**^**−1**^	**43.6 ppb**	**↓**
PM_10_	**VA**	** − 0.26**	** − 5.81**	** − 0.35 µg m**^**−3**^** year**^**−1**^	**29.3 µg m**^**−3**^	**↓**
O_3_	ML	0.00	− 0.01	− 0.01 µg m^**−**3^ year^**−**1^	51.8 µg m^**−**3^	↔
ΔO_3_	-	0.05	0.96	0.13 µg m^**−**3^ year^**−**1^	− 10.49 µg m^**−**3^	↔

The level of atmospheric pollutants in the city of Rome is mainly related to the emissions from vehicular traffic (Cattani et al. 2010), and, to a lesser extent, to domestic heating and transport of particulate matter from other areas. The contribution of biomass burning for domestic heating is low (Perrino et al. 2019), whilst the dust advection events, although with a significant impact on the atmospheric content of both coarse (PM_10_) and fine (PM_2.5_) particulate matters, are typically limited in time (Gobbi et al. 2006). Furthermore, it should be noted that in 2020, Italy was subject to the lockdown restrictions imposed to face the COVID-19 pandemic. This led to a decrease in the emissions of primary pollutants, due to the temporary shutdown of numerous economical activities and a strong reduction in vehicular traffic. As demonstrated by Campanelli et al. (2021) and Bassani et al. (2021), in the area under examination, the air quality was affected by these policies, with a general reduction in the atmospheric concentration of NO_2_, C_6_H_6_ and PM_10_ and an increase in the in situ O_3_ concentration.

The reduction in the C_6_H_6_ (Fig. 4a) and SO_2_ (Fig. 4b) concentrations is mainly attributable to the renewal of the vehicle fleet, with cars’ fuel containing a lower quantity of benzene (ACI 2021; Piras et al. 2019). Except for rare cases, since 2003, the concentration of C_6_H_6_ is always below 4 µg m^−3^, whilst SO_2_ averages are below 2.5 µg m^−3^. The national emissions inventory (ISPRA 2021a, 2021b) estimated, between 1990 and 2019, a decline in SO_2_ emissions from 1784 to 105 Gg, with the almost total elimination of emissions due to road traffic emissions. The SO_2_ concentration peaks detected in February 2000 and October 2012 can be linked to the long-range transport of volcanic ash caused by eruptions of Etna (Pardini et al. 2017), whilst the peak recorded in January 2016 is due to fires of waste and farmlands occurred in the surrounding of the VA measurement station. The SK test shows during all seasons a significant decline for C_6_H_6_ (τ =  − 0.73, Z =  − 16.46 and slope − 0.12 µg m^−3^ year^−1^) and a rather limited one for SO_2_ (τ =  − 0.46, Z =  − 10.39 and slope − 0.09 µg m^−3^ year^−1^). It is worth noticing, however, that a linear trend may not accurately describe the decrease in the amounts of C_6_H_6_ and SO_2_, which instead shows a plateau in recent years. A more meticulous representation will be the subject of future studies.

The CO concentration, depicted in Fig. 4c, has a significant negative trend throughout the year, as highlighted also by the SK test results (τ =  − 0.75, Z =  − 17.05 and slope − 0.02 mg m^−3^ year^−1^). CO assumes average values between 0.4 and 0.8 mg m^−3^, in line with the reduction of the national CO emissions in the period 1990–2019 (from 6797 to 2062 Gg) (ISPRA 2021a, 2021b) when the road traffic emissions decreased by 92%.

The NO_x_ trend (Fig. 4d) can be considered as a tracer of the evolution of the vehicle fleet in urban areas. The reduction of NO and NO_2_, more pronounced from 2018, could be attributable to the progressive tendency towards the reduction of the petrol vehicles, in favour of hybrid automobiles, as confirmed by the analysis of national emissions of NO_x_ which, in the period 1990–2019, decreased from 2125 to 627 Gg (ISPRA 2021a, 2021b). National road traffic emissions decreased by 75% in the period 1990–2019. Furthermore, the year 2018 coincides with the national transposition of the European Directive 2016/2284/EU on the reduction of national emissions of atmospheric pollutants (EU 2016), which has amongst the main objectives the reduction of annual anthropogenic emissions of NO_2_ promoting electrification, particularly in the transport sector. NO_x_ assumes average values between 30 µg m^−3^ in summer and 200 µg m^−3^ in winter, whilst NO monthly averages are always below 65 µg m^−3^. The different reduction rates of NO_x_ (τ =  − 0.45, Z =  − 10.30 and slope − 1.28 µg m^−3^ year^−1^) and NO (τ =  − 0.45, Z =  − 10.17 and slope − 1.38 µg m^−3^ year^−1^) compared to NO_2_ (τ =  − 0.36, Z =  − 8.14 and slope − 0.60 µg m^−3^ year^−1^) are attributable to the increased NO_2_/NO_x_ ratio in vehicular exhaust: diesel vehicles emit more NO_2_ in proportion to NO_x_. As suggested by Alvarez et al. (2008), the number of diesel cars in circulation (increasing in Italy until 2017), together with the particulate filters and oxidation catalysts used in modern diesel engines, is probably responsible for the lower NO_2_ decreases in road environments. As expected, the reduction in emissions means that the trend is negative in all seasons.

The O_3_ (Fig. 4e) concentration (average values between 10 µg m^−3^ and 100 µg m^−3^) slightly increases over the years, as demonstrated by the values of Z = 2.46, τ = 0.15 and slope 0.15 µg m^−3^ year^−1^ obtained by the SK test, with the inter-seasonal fluctuations ascribable to the natural variations. The seasonal analysis shows that the trend is positive in spring and winter, negative in summer and insignificant in autumn. This behaviour might be partly justified by the reduced concentrations of NO_x_ that, during the summer, leads to a weakening that does not allow for the photochemical reactions and that, in the winter, reduces the titration of O_3_ by the NO. Positive trends agree with findings by Tarasick et al. (2019), who studied many European urban and rural sites, although the growth rate in Rome is higher (0.15 µg m^−3^ year^−1^) than the European urban average (about 0.10 µg m^−3^ year^−1^).

Vertical transport of tropospheric ozone can additionally influence boundary layer processes and surface ozone concentrations. In order to evaluate this effect, the data collected by the VA urban station were compared with those measured in the rural area of ML. As shown in Fig. A1a, at ML, the average monthly concentrations of O_3_ are always higher than in the city centre. Common peaks can be observed, both in rural and urban environments, in correspondence with the hottest heatwaves occurred in the Italian peninsula during the summers of 2003, 2011 and 2012, as outlined also by Tarasick et al. (2019) and Gaudel et al. (2018). This underlines the importance of the contribution of tropospheric ozone, in addition to the photochemical production, in the processes governing the boundary layer. The application of the SK test shows that, contrary to Rome downtown, a statistically significant annual trend is not detectable at ML (τ = 0.00, Z =  − 0.01 and slope − 0.01 µg m^−3^ year^−1^) and that only during summer a decreasing trend (τ =  − 0.40, Z = 2.54 and slope − 4.80 µg m^−3^ year^−1^) is observable, perhaps suggesting different photochemical regimes and dynamics. This aspect will be investigated in a future dedicated study. The average values are typically below 50 µg m^−3^ with peaks up to 150 µg m^−3^ and a seasonal cycle linked to meteorological variables and to volume of urban traffic. Generally, the highest PM_10_ concentrations are measured in winter, when the lowest levels of solar radiation affect the structure of the atmospheric boundary layer, limiting convection and turbulence and leading to stagnation episodes associated with pollutant accumulation in the lower layers of the atmosphere due to poor dispersion conditions. Peaks in the average monthly concentrations are attributable to medium-long-range transport phenomena and are typically measured in the case of desert dust outbreaks (Gobbi et al. 2017). The national emissions inventory depicts a decreasing trend for PM_10_ in the period 1990–2019, from 293 to 172 Gg (ISPRA 2021a, 2021b). Vehicular traffic emissions decreased by 66% due to the introduction of European Directives controlling and limiting PM emissions at the car exhaust pipe. This is experimentally confirmed by the observed downward temporal trend in PM_10_ (Fig. 4f) (during all seasons except winter, when no trend is evident), although the values assumed by Z (− 5.81), τ (− 0.26) and the slope (− 0.35 µg m^−3^ year^−1^) show a slow reduction and a rather weak monotonic pattern. Consequences on other research fields are expected, for example Fountoulakis et al. (2021) found an increase in ultraviolet radiation on the ground not linked to changes in ozone.

Finally, the temporal trend of the potential ozone O_x_ was also evaluated (Fig. 5c) as the sum of NO_2_ and O_3_. For this reason, the concentrations of NO_2_ (Fig. 5a) and O_3_ (Fig. 5b) have been expressed in ppb (using coefficients referring to the air temperature of 25 °C and to the pressure of 1 atm, equal to 0.53 and 0.50 for NO_2_ and O_3_, respectively). The potential ozone depicts a statistically significant downward temporal trend assuming (Z =  − 5.51 and τ =  − 0.25). The slope is − 0.28 ppb year^−1^, i.e. shows an annual reduction between that of NO_2_ (− 0.33 ppb year^−1^) and O_3_ (0.07 ppb year^−1^). Seasonal analysis shows downward trends in summer (τ =  − 0.50, Z =  − 4.30 and slope − 2.16 ppb year^−1^) and autumn (τ =  − 0.39, Z =  − 3.39 and slope − 1.18 ppb year^−1^). On the contrary, no statistically significant temporal variations are observed during spring and winter. This behaviour will be addressed in more depth in future studies.

Overall, the air quality trends found here agree with the results by Tomassetti et al. (2020), who investigated the impact of mobility policies on air quality in 14 major Italian cities, including Rome, in the period 2006–2016. They found a general reduction in PM_10_ and NO_2_ but with a still high number of exceedances, stressing the importance of studying climatic conditions as well. Gualtieri et al. (2014) examined the annual trends of the main atmospheric pollutant concentrations in Florence (Italy) based on surface urban observations. In agreement with our results, they found a significant decrease for primary pollutants, whilst this was poorly significant for secondary species despite a remarkable reduction in their precursor emissions. Several scientific studies have shown that in Europe, i.e. even in regions with climatic conditions different from Italy, the situation is quite similar. For example, Zara et al. (2021) examined the reductions in nitrogen oxides over the Netherlands between 2005 and 2018, using satellite observations and ground-based measurements. They observed a 30% reduction in NO_2_ concentration, with a stronger reduction in NO_x_ than in NO_2_, accompanied by a long-term increase in O_3_, suggesting changes in the chemical regimes and NO_x_ lifetime. Anttila and Tuovinen (2010) found, in the urban Finnish environment, a net decline of surface SO_2_, CO and NO_x_ concentrations, a limited reduction in NO_2_ concentrations and a positive trend for O_3_. In fact, for pollutants generated through photochemical reactions, as for O_3_, the concentrations increase with increasing solar radiation and temperature, causing a seasonal and daily cyclicality with maxima concentrations in summer and in the hours immediately following those of maximum solar radiation. Moreover, the O_3_ can be dispersed by the wind from urban to rural areas, where fewer pollution levels slow down the ozone reactions, making the molecule more stable. The analysis of the data from the rural ML station, however, also highlights the importance of vertical transport of tropospheric ozone in the boundary layer and close to the ground level. The overall trends estimated in this work agree with the results by Cattani et al. (2010) who applied the SK test to atmospheric pollutants measured in Rome in the period 1999–2008. Our results show that when expanding the time range, the tendencies do not vary significantly and that NO_2_ has started to show a significant negative trend only in recent years, as shown in Fig. 4.

## Conclusions

A meteorological and air quality study has been carried out through in situ, ground-based observations collected during the last two decades in different sites located in the urban area of Rome (Italy) and its coastal surroundings. One of the greatest difficulties in these studies is the lack of data series sufficiently long and reliable. To overcome this limit, datasets covering the period 2000–2020 provided by different national research organisations are used. The data were pre-processed to eliminate gross errors and to verify the homogeneity of the time series. The Craddock test is applied, founding two breakpoints for the RH series measured in one of the urban stations, which was excluded from the evaluation of temporal trends. The Seasonal Kendall test is applied to verify the presence of statistically significant temporal trends.

The meteorological data show substantial positive trends for average, minimum and maximum air temperature, mixing ratio and heat index both in the urban and coastal environment. Otherwise, total precipitations have a constant behaviour in the urban area. These data represent an important basis for understanding the seasonality and temporal variability of a complex urban environment, to support evidence-based policies, and as a reference for the comparison of numerical simulations aimed at mitigating climate change or the UHI phenomenon.

The air quality data depict a negative trend for C_6_H_6_, SO_2_, CO, NO_x_, NO and O_x_. For secondary pollutants, i.e. NO_2_ and O_3_, the progress in air quality improvement has been quite limited. In fact, NO_2_ has a statistically significant decreasing trend, which is more pronounced from 2018 when, thanks to the ecological transition policies, the number of diesel cars has decreased, promoting the use of hybrid vehicles. The concentration of surface O_3_ is increasing over the years in Rome downtown, as a likely effect of the NOx reduction in the city centre. On the contrary, in the rural area adjacent to Rome, the ozone does not show a statistically significant trend. For PM_10_, the trend is slightly decreasing but, in this case, the analysis is made more complex by the coexistence of local sources and particulate matter advection from the sea and desert regions.

The results allow us to draw the following conclusions and recommendations, useful for decision-makers and stakeholders:

(i) In the city of Rome, i.e. in a metropolis with a well-established historical development and subjected to a rather limited downtown transformation and expansion, growing thermal discomfort due to rising temperatures—as a consequence of climate change that also affects this region, as confirmed by other studies—is observed. This means that an increase in energy consumption for cooling is expected and, consequently, this will lead to a further worsening of urban thermal comfort. Moreover, the statistically significant positive trends of minimum, average and maximum temperature and mixing ratio highlight a potential negative effect on buildings, with important consequences on the conservation and safeguarding of cultural heritage;
(ii) The air quality level is improving, even if the tendency for NO_2_ and O_3_ is weak. Therefore, the regional and national regulations adopted in recent years are working and it is necessary to intensify the reduction of local emissions as much as possible through environmental protection policies and limiting the use of cars, supporting the renewal of the vehicle fleet, and encouraging the transition to hybrid and electric automobiles.

It can be concluded that local-scale actions are showing encouraging results, but a common and global commitment is needed to limit global warming, applying the appropriate mitigation strategies.

Finally, this study highlights the importance of maintaining active long-term networks, capable of collecting quality-checked data, with harmonised characteristics and locations that can be used to build datasets for the study of past and present atmospheric composition and, therefore, to predict future scenarios.

## Supplementary Information

Below is the link to the electronic supplementary material.

Supplementary file1 (DOCX 368 kb)

## Data Availability

The datasets analysed during the current study are available in the following repositories: https://www.idrografico.regione.lazio.it (meteorological data for RM and IS stations), http://www.arsial.it/arsial/ (meteorological data for RL), http://www.arpalazio.net/main/aria/sci/basedati/chimici/chimici.php (air quality for VA) and 10.1594/PANGAEA.876108 (ozone for ML). Meteorological data for CR, provided by CREA, are available at https://www.crea.gov.it/home until 2012. For the subsequent period, data are not publicly available and can be requested to CREA. Data provided by ENAV for the FA station are not publicly available and can be requested to Italian Air Force Meteorological Service.

## Code availability

The codes used for the processing of data can be provided on request to the corresponding author.
