# Supplementary material for: Analysis of two-decade meteorological and air quality trends in Rome (Italy)
Source: Theor Appl Climatol. 2022 Apr 14;149(1-2):291–307. doi: 10.1007/s00704-022-04047-y (PMC9008384; doi:10.1007/s00704-022-04047-y)
Supplement: Supplementary file 1 — Supplementary file1 (DOCX 368 kb) [file 704_2022_4047_MOESM1_ESM.docx]

# Appendix A

| **Variable** | **Site** | **Monthly average** | | | | | | | | | | | | **Annual average** |  |
| --- | --- | --- | --- | --- | --- | --- | --- | --- | --- | --- | --- | --- | --- | --- | --- |
|  |  | Jan | Feb | Mar | Apr | May | Jun | Jul | Aug | Sep | Oct | Nov | Dec |  |  |
| **T_ave_**  **(°C)** | CR | 8.6 | 9.6 | 12.3 | 15.5 | 19.7 | 23.8 | 26.4 | 26.3 | 22.0 | 18.2 | 13.6 | 9.7 | 17.1 |  |
|  | RM/RL | 9.1 | 10.1 | 12.8 | 16.1 | 20.0 | 24.4 | 27.0 | 27.1 | 22.9 | 19.0 | 14.3 | 10.1 | 17.7 |  |
|  | FA/IS | 8.2 | 8.8 | 11.2 | 14.2 | 18.0 | 22.2 | 24.6 | 24.9 | 21.2 | 17.4 | 13.3 | 9.4 | 16.1 |  |
| **T_min_**  **(°C)** | CR | 5.3  (-1.9, 2000) | 6.1  (-4.6, 2018) | 8.5  (-2.6, 2005) | 11.3  (0.7, 2003) | 15.0  (9.2, 2004) | 19.0  (10.5, 2010) | 21.5  (5.2, 2011) | 21.7  (14.4, 2016) | 17.9  (9.0, 2008) | 14.3  (5.4, 2009) | 10.2  (0.4, 2005) | 6.5  (-1.2, 2007) | 13.1 |  |
|  | RM/RL | 5.1  (2.9, 2000) | 5.8  (2.2, 2003) | 8.3  (6.9, 2005) | 11.1  (9.7, 2005) | 14.9  (11.9, 2019) | 19.0  (17.3, 2006) | 21.5  (19.9, 2014) | 21.6  (19.6, 2005) | 17.8  (15.8, 2001) | 14.2  (12.2, 2009) | 10.1  (7.8, 2007) | 6.2  (4.0, 2001) | 13.0 |  |
|  | FA/IS | -1.5  (-5.0, 2004) | -1.6  (-5.0, 2018) | 0.9  (-4.2, 2005) | 3.9  (1.0, 2003) | 8.1  (5.0, 2019) | 11.9  (9.0, 2005) | 15.1  (12.0, 2000) | 15.7  (14.0, 2002) | 11.0  (8.0, 2007) | 7.3  (3.0, 2009) | 2.7 (0.0, 2005) | -0.9  (-4.2, 2010) | 6.0 |  |
| **T_max_**  **(°C)** | CR | 12.3  (18.3, 2006) | 13.5  (21.6, 2019) | 16.4  (25.4, 2001) | 19.8  (29.4, 2013) | 24.1  (33.5, 2008) | 28.7  (36.6, 2006) | 31.3  (39.1, 2005) | 31.3  (38.8, 2003) | 26.7  (34.8, 2015) | 22.5  (30.9, 2000) | 17.3  (23.5, 2008) | 13.0 (20.4, 2004) | 21.2 |  |
|  | RM/RL | 13.1  (15.9, 2018) | 14.4  (17.6, 2020) | 17.4  (21.1, 2012) | 21.0  (24.1, 2018) | 25.1  (27.5, 2009) | 29.9  (33.4, 2003) | 32.4  (35.2, 2015) | 32.7  (35.2, 2003) | 28.0  (31.2, 2011) | 23.7  (26.1, 2019) | 18.4  (20.4, 2014) | 14.1  (16.0, 2019) | 22.5 |  |
|  | FA/IS | 16.7  (20.0, 2018) | 17.6  (22.0, 2014) | 20.4  (24.0, 2016) | 23.8  (28.0, 2013) | 28.3  (33.0, 2008) | 32.1  (36.0, 2019) | 33.2  (34.0, 2005) | 33.9  (36.0, 2003) | 30.3  (33.1, 2015) | 26.0  (30.0, 2000) | 22.3  (26.1, 2004) | 18.1  (20.0, 2014) | 25.2 |  |
| **MR**  **(g kg^-1^)** | RM/RL | 5.6 | 5.6 | 6.4 | 7.8 | 9.4 | 11.5 | 12.9 | 13.1 | 11.7 | 9.9 | 8.0 | 5.9 | 9.0 |  |
|  | FA/IS | 5.2 | 5.3 | 6.2 | 7.4 | 9.1 | 11.5 | 13.1 | 13.4 | 11.1 | 9.4 | 7.5 | 5.7 | 8.7 |  |
| **TP**  **(mm)** | CR | 58.2 | 57.0 | 57.9 | 52.8 | 45.6 | 21.9 | 19.5 | 27.7 | 58.7 | 64.4 | 103.2 | 72.0 | 53.2 |  |
|  | RM/RL | 69.6 | 69.0 | 66.4 | 58.7 | 64.0 | 27.6 | 25.4 | 28.8 | 74.8 | 79.8 | 126.7 | 89.5 | 65.0 |  |
|  | FA/IS | 36.5 | 43.1 | 39.9 | 26.4 | 25.0 | 15.9 | 7.2 | 12.2 | 48.8 | 61.2 | 78.8 | 45.9 | 36.7 |  |
| **HI**  **(°C)** | RM/RL | 6.2 | 7.3 | 10.5 | 14.8 | 18.6 | 23.2 | 26.0 | 26.1 | 22.0 | 17.5 | 12.1 | 7.0 | 15.9 |  |
|  | FA/IS | 4.4 | 5.2 | 8.3 | 11.9 | 16.4 | 20.9 | 23.3 | 23.5 | 19.8 | 15.7 | 10.9 | 5.9 | 13.8 |  |

**Table A1** Values of monthly and annual average meteorological parameters in the period 2000-2020 for urban (CR and RM/RL) and coastal (FA/IS) environments. The absolute minimum and maximum temperatures (T_min_ and T_max_) and the year of occurrence are indicated in brackets for each month

| **Variable** | **Site** | **Season** | **τ** | **Z** | **Slope** | **Intercept** | **Trend** |
| --- | --- | --- | --- | --- | --- | --- | --- |
| **T_ave_** | CR | **spring** | **0.34** | **2.83** | **0.08 °C year^-1^** | **14.5 °C** | **↑** |
|  |  | **summer** | **0.25** | **2.06** | **0.09 °C year^-1^** | **24.8 °C** | **↑** |
|  |  | autumn | 0.22 | 1.86 | 0.05 °C year^-1^ | 17.5 °C | ↔ |
|  |  | winter | 0.23 | 1.96 | 0.09 °C year^-1^ | 8.3 °C | ↔ |
|  | FA/IS | spring | 0.19 | 1.56 | 0.05 °C year^-1^ | 13.7 °C | ↔ |
|  |  | **summer** | **0.32** | **2.74** | **0.08 °C year^-1^** | **23.3 °C** | **↑** |
|  |  | **autumn** | **0.24** | **2.09** | **0.05 °C year^-1^** | **16.9 °C** | **↑** |
|  |  | winter | 0.10 | 0.78 | 0.05 °C year^-1^ | 8.4 °C | ↔ |
|  | RM/RL | spring | 0.19 | 1.64 | 0.05 °C year^-1^ | 15.4 °C | ↔ |
|  |  | **summer** | **0.32** | **2.81** | **0.10 °C year^-1^** | **25.3 °C** | **↑** |
|  |  | **autumn** | **0.24** | **2.03** | **0.05 °C year^-1^** | **18.2 °C** | **↑** |
|  |  | winter | 0.20 | 1.66 | 0.07 °C year^-1^ | 9.2 °C | ↔ |
| **T_max_** | CR | spring | 0.08 | 0.65 | 0.03 °C year^-1^ | 19.3 °C | ↔ |
|  |  | summer | 0.15 | 1.30 | 0.03 °C year^-1^ | 30.6 °C | ↔ |
|  |  | autumn | 0.15 | 1.24 | 0.02 °C year^-1^ | 22.2 °C | ↔ |
|  |  | winter | 0.27 | 1.11 | 0.05 °C year^-1^ | 12.7 °C | ↔ |
|  | FA/IS | spring | 0.19 | 1.60 | 0.07 °C year^-1^ | 22.8 °C | ↔ |
|  |  | summer | 0.20 | 1.84 | 0.05 °C year^-1^ | 32.7 °C | ↔ |
|  |  | autumn | 0.10 | 0.90 | 0.00 °C year^-1^ | 26.0 °C | ↔ |
|  |  | winter | 0.15 | 1.33 | 0.00 °C year^-1^ | 17.0 °C | ↔ |
|  | RM/RL | spring | 0.19 | 1.65 | 0.07 °C year^-1^ | 20.4 °C | ↔ |
|  |  | **summer** | **0.38** | **3.35** | **0.12 °C year^-1^** | **30.4 °C** | **↑** |
|  |  | **autumn** | **0.28** | **2.42** | **0.07 °C year^-1^** | **22.9 °C** | **↑** |
|  |  | **winter** | **0.27** | **2.29** | **0.10 °C year^-1^** | **12.7 °C** | **↑** |
| **T_min_** | CR | **spring** | **0.33** | **2.87** | **0.11 °C year^-1^** | **10.3 °C** | **↑** |
|  |  | **summer** | **0.41** | **3.53** | **0.13 °C year^-1^** | **19.2 °C** | **↑** |
|  |  | **autumn** | **0.33** | **2.87** | **0.11 °C year^-1^** | **13.2 °C** | **↑** |
|  |  | **winter** | **0.26** | **2.22** | **0.11 °C year^-1^** | **4.9 °C** | **↑** |
|  | FA/IS | spring | 0.12 | 1.08 | 0.00 °C year^-1^ | 3.9 °C | ↔ |
|  |  | **summer** | **0.23** | **2.06** | **0.06 °C year^-1^** | **14.3 °C** | **↑** |
|  |  | autumn | 0.08 | 0.75 | 0.00 °C year^-1^ | 8.0 °C | ↔ |
|  |  | winter | 0.16 | 1.42 | 0.08 °C year^-1^ | -1.8 °C | ↔ |
|  | RM/RL | spring | -0.06 | -0.46 | -0.01 °C year^-1^ | 11.3 °C | ↔ |
|  |  | summer | 0.18 | 1.59 | 0.05 °C year^-1^ | 20.4 °C | ↔ |
|  |  | autumn | 0.13 | 1.04 | 0.02 °C year^-1^ | 13.7 °C | ↔ |
|  |  | winter | 0.06 | 0.47 | 0.04 °C year^-1^ | 5.4 °C | ↔ |
| **MR** | FA/IS | spring | 0.07 | 0.52 | 0.00 g kg^-1^ year^-1^ | 7.5 g kg^-1^ | ↔ |
|  |  | summer | 0.20 | 1.69 | 0.04 g kg^-1^ year^-1^ | 12.5 g kg^-1^ | ↔ |
|  |  | **autumn** | **0.26** | **2.22** | **0.05 g kg^-1^ year^-1^** | **9.0 g kg^-1^** | **↑** |
|  |  | winter | 0.08 | 0.65 | 0.02 g kg^-1^ year^-1^ | 5.2 g kg^-1^ | ↔ |
|  | RM/RL | **spring** | **0.33** | **2.12** | **0.05 g kg^-1^ year^-1^** | **7.1 g kg^-1^** | **↑** |
|  |  | **summer** | **0.59** | **3.81** | **0.23 g kg^-1^ year^-1^** | **9.4 g kg^-1^** | **↑** |
|  |  | **autumn** | **0.44** | **3.04** | **0.15 g kg^-1^ year^-1^** | **7.8 g kg^-1^** | **↑** |
|  |  | **winter** | **0.33** | **2.12** | **0.07 g kg^-1^ year^-1^** | **4.7 g kg^-1^** | **↑** |
| **TP** | CR | spring | -0.15 | -1.30 | -1.40 mm year^-1^ | 60.0 mm | ↔ |
|  |  | summer | 0.09 | 0.72 | 0.10 mm year^-1^ | 14.8 mm | ↔ |
|  |  | autumn | -0.02 | -0.13 | -0.34 mm year^-1^ | 61.9 mm | ↔ |
|  |  | winter | -0.07 | -0.59 | -0.72 mm year^-1^ | 56.4 mm | ↔ |
|  | FA/IS | **spring** | **0.28** | **2.44** | **1.7 mm year^-1^** | **2.8 mm** | **↑** |
|  |  | **summer** | **0.34** | **3.06** | **0.22 mm year^-1^** | **-0.7 mm** | **↑** |
|  |  | **autumn** | **0.32** | **2.76** | **3.02 mm year^-1^** | **13.3 mm** | **↑** |
|  |  | winter | 0.18 | 1.59 | 1.47 mm year^-1^ | 12.0 mm | ↔ |
|  | RM/RL | spring | 0.00 | 0.00 | 0.20 mm year^-1^ | 54.5 mm | ↔ |
|  |  | summer | 0.13 | 1.07 | 0.45 mm year^-1^ | 14.1 mm | ↔ |
|  |  | autumn | 0.04 | 0.33 | 1.32 mm year^-1^ | 67.7 mm | ↔ |
|  |  | winter | 0.00 | 0.00 | -0.12 mm year^-1^ | 59.6 mm | ↔ |
| **HI** | FA/IS | spring | 0.18 | 1.56 | 0.04 °C year^-1^ | 11.5 °C | ↔ |
|  |  | **summer** | **0.35** | **3.00** | **0.08 °C year^-1^** | **22.0 °C** | **↑** |
|  |  | **autumn** | **0.26** | **2.21** | **0.05 °C year^-1^** | **15.3 °C** | **↑** |
|  |  | winter | 0.11 | 0.91 | 0.06 °C year^-1^ | 4.6 °C | ↔ |
|  | RM/RL | spring | 0.17 | 1.06 | 0.05 °C year^-1^ | 13.8 °C | ↔ |
|  |  | **summer** | **0.46** | **2.97** | **0.12 °C year^-1^** | **23.6 °C** | **↑** |
|  |  | **autumn** | **0.36** | **2.45** | **0.09 °C year^-1^** | **16.1 °C** | **↑** |
|  |  | winter | 0.17 | 1.06 | 0.12 °C year^-1^ | 5.2 °C | ↔ |

**Table A2** Results of Seasonal Kendall test for the meteorological data collected both at the urban (CR, RM/RL) and coastal (FA/IS) sites. For each season, Kendall correlation coefficient and slope are expressed as yearly rates of variation. In the trend column, ↑ (↓) indicates a positive (negative) statistically significant trend, while ↔ depicts no statistically significant trend. The seasons with statistically significant trends are in bold

| **Variable** | **Site** | **Season** | **τ** | **Z** | **Slope** | **Intercept** | **Trend** |
| --- | --- | --- | --- | --- | --- | --- | --- |
| **C_6_H_6_** | VA | **spring** | **-0.78** | **-6.76** | **-0.38 μg m^-3^ year^-1^** | **2.0 μg m^-3^** | **↓** |
|  |  | **summer** | **-0.77** | **-6.62** | **-0.36 μg m^-3^ year^-1^** | **1.8 μg m^-3^** | **↓** |
|  |  | **autumn** | **-0.84** | **-7.30** | **-0.51 μg m^-3^ year^-1^** | **2.8 μg m^-3^** | **↓** |
|  |  | **winter** | **-0.71** | **-6.09** | **-0.57 μg m^-3^ year^-1^** | **3.7 μg m^-3^** | **↓** |
| **SO_2_** | VA | **spring** | **-0.41** | **-3.55** | **-0.27 μg m^-3^ year^-1^** | **1.6 μg m^-3^** | **↓** |
|  |  | **summer** | **-0.58** | **-4.87** | **-0.33 μg m^-3^ year^-1^** | **1.8 μg m^-3^** | **↓** |
|  |  | **autumn** | **-0.46** | **-3.95** | **-0.25 μg m^-3^ year^-1^** | **1.6 μg m^-3^** | **↓** |
|  |  | **winter** | **-0.58** | **-5.02** | **-0.50 μg m^-3^ year^-1^** | **2.4 μg m^-3^** | **↓** |
| **CO** | VA | **spring** | **-0.82** | **-7.03** | **-0.08 mg m^-3^ year^-1^** | **0.6 mg m^-3^** | **↓** |
|  |  | **summer** | **-0.79** | **-6.84** | **-0.07 mg m^-3^ year^-1^** | **0.5 mg m^-3^** | **↓** |
|  |  | **autumn** | **-0.83** | **-7.16** | **-0.12 mg m^-3^ year^-1^** | **0.8 mg m^-3^** | **↓** |
|  |  | **winter** | **-0.81** | **-7.03** | **-0.11 mg m^-3^ year^-1^** | **0.9 mg m^-3^** | **↓** |
| **NO_x_** | VA | **spring** | **-0.38** | **-3.28** | **-4.70 μg m^-3^ year^-1^** | **60.9 μg m^-3^** | **↓** |
|  |  | **summer** | **-0.46** | **-3.95** | **-2.82 μg m^-3^ year^-1^** | **43.1 μg m^-3^** | **↓** |
|  |  | **autumn** | **-0.45** | **-3.81** | **-5.60 μg m^-3^ year^-1^** | **81.9 μg m^-3^** | **↓** |
|  |  | **winter** | **-0.41** | **-3.55** | **-9.93 μg m^-3^ year^-1^** | **124.6 μg m^-3^** | **↓** |
| **NO** | VA | **spring** | **-0.45** | **-3.89** | **-1.04 μg m^-3^ year^-1^** | **11.3 g m^-3^** | **↓** |
|  |  | **summer** | **-0.34** | **-2.88** | **-0.39 μg m^-3^ year^-1^** | **6.2 μg m^-3^** | **↓** |
|  |  | **autumn** | **-0.46** | **-3.95** | **-2.26 μg m^-3^ year^-1^** | **24.1 μg m^-3^** | **↓** |
|  |  | **winter** | **-0.44** | **-3.81** | **-4.91 μg m^-3^ year^-1^** | **46.7 μg m^-3^** | **↓** |
| **NO_2_** | VA | **spring** | **-0.38** | **-3.28** | **-2.66 μg m^-3^ year^-1^** | **40.3 μg m^-3^** | **↓** |
|  |  | **summer** | **-0.34** | **-2.88** | **-0.39 μg m^-3^ year^-1^** | **6.3 μg m^-3^** | **↓** |
|  |  | **autumn** | **-0.28** | **-2.34** | **-2.00 μg m^-3^ year^-1^** | **43.5 μg m^-3^** | **↓** |
|  |  | **winter** | **-0.35** | **-3.01** | **-2.70 μg m^-3^ year^-1^** | **53.3 μg m^-3^** | **↓** |
| **O_3_** | VA | **spring** | **0.31** | **2.61** | **1.53 μg m^-3^ year^-1^** | **44.1 μg m^-3^** | **↑** |
|  |  | **summer** | **-0.26** | **-2.21** | **-1.51 μg m^-3^ year^-1^** | **66.5 μg m^-3^** | **↓** |
|  |  | autumn | 0.09 | 0.74 | 0.53 μg m^-3^ year^-1^ | 28.5 μg m^-3^ | ↔ |
|  |  | **winter** | **0.31** | **2.61** | **1.41 μg m^-3^ year^-1^** | **16.1 μg m^-3^** | **↑** |
| **O_x_** | VA | spring | -0.21 | -1.83 | -0.74 ppb year^-1^ | 44.5 ppb | ↔ |
|  |  | **summer** | **-0.50** | **-4.30** | **-2.16 ppb year^-1^** | **561.4 ppb** | **↓** |
|  |  | **autumn** | **-0.39** | **-3.39** | **-1.18 ppb year^-1^** | **38.7 ppb** | **↓** |
|  |  | winter | -0.15 | -1.27 | -0.62 ppb year^-1^ | 36.3 ppb | ↔ |
| **PM_10_** | VA | **spring** | **-0.39** | **-3.35** | **-1.66 μg m^-3^ year^-1^** | **27.8 μg m^-3^** | **↓** |
|  |  | **summer** | **-0.63** | **-5.42** | **-1.96 μg m^-3^ year^-1^** | **28.8 μg m^-3^** | **↓** |
|  |  | **autumn** | **-0.40** | **-3.41** | **-1.64 μg m^-3^ year^-1^** | **28.9 μg m^-3^** | **↓** |
|  |  | winter | -0.13 | -1.06 | -0.67 μg m^-3^ year^-1^ | 32.1 μg m^-3^ | ↔ |
| **O_3_** | ML | spring | 0.14 | 0.85 | 0.54 μg m^-3^ year^-1^ | 56.2 μg m^-3^ | ↔ |
|  |  | **summer** | **-0.40** | **2.54** | **-4.80 μg m^-3^ year^-1^** | **82.6 μg m^-3^** | **↓** |
|  |  | autumn | 0.14 | 0.85 | 0.40 μg m^-3^ year^-1^ | 37.9 μg m^-3^ | ↔ |
|  |  | winter | 0.27 | 1.70 | 1.21 μg m^-3^ year^-1^ | 27.6 μg m^-3^ | ↔ |
| **ΔO_3_** | **-** | spring | 0.05 | 0.21 | 0.33 μg m^-3^ year^-1^ | -11.4 μg m^-3^ | ↔ |
|  |  | summer | 0.30 | 1.91 | 1.96 μg m^-3^ year^-1^ | -12.8 μg m^-3^ | ↔ |
|  |  | autumn | 0.05 | 0.21 | 0.46 μg m^-3^ year^-1^ | -7.5 μg m^-3^ | ↔ |
|  |  | winter | -0.08 | -0.42 | -0.32 μg m^-3^ year^-1^ | -11.8 μg m^-3^ | ↔ |

**Table A3** Results of Seasonal Kendall test for in-situ air pollutants. For each season, Kendall correlation coefficient and slope are expressed as yearly rates of variation. Note that ΔO_3_ is defined as the difference between urban (VA) and rural (ML) concentrations and, together with O_3_ at ML, refer to the period 2000-2014. In the trend column, ↑ (↓) indicates a positive (negative) statistically significant trend, while ↔ depicts no statistically significant trend. The seasons with statistically significant trends are in bold

**
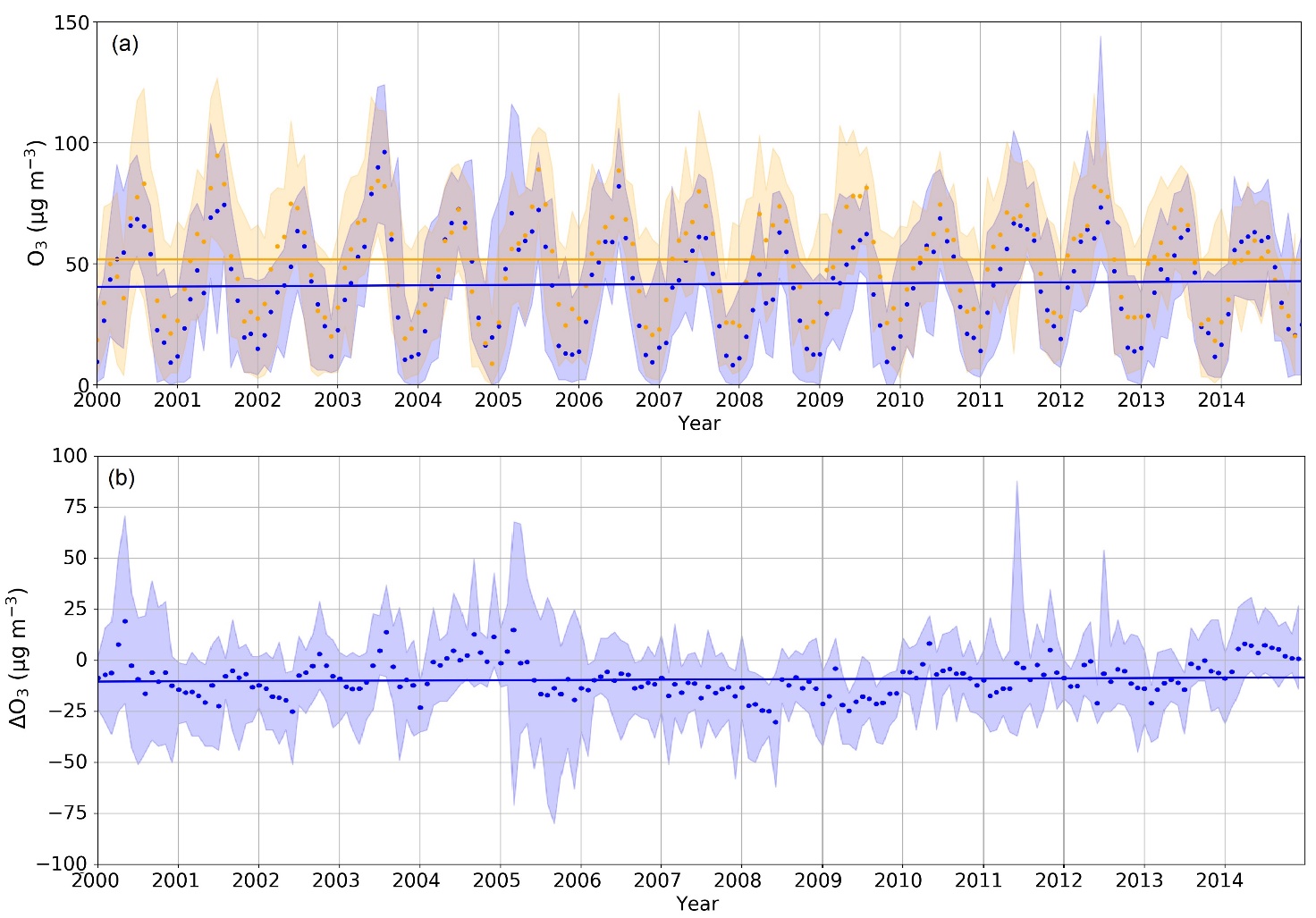
**

**Fig. A1** Trend of monthly average in-situ concentrations of (a) O_3_ measured at VA (blue line) and ML (orange line), and (b) ΔO_3,_ defined as the difference between urban (VA) and rural (ML) O_3_ levels over the period 2000-2014. The shaded regions represent minima and maxima values. The solid lines depict the Kendall-Theil Robust Line, computed considering the slope and the intercept of the SK test for each parameter
